# Milk of calcium in scleromyositis

**DOI:** 10.1093/rheumatology/keaf568

**Published:** 2025-10-25

**Authors:** Chiara Giraudo, Francesco Zulian, Elisabetta Zanatta

**Affiliations:** Unit of Advanced Clinical and Translational Imaging, Department of Cardiac, Thoracic, Vascular Sciences and Public Health—DCTV, University of Padova, Padova, Italy; Pediatric Rheumatology Unit, Department of Women’s and Children’s Health, University of Padova, Padova, Italy; Rheumatology Unit, Department of Medicine—DIMED, University of Padova, Padova, Italy

A 15-year-old female with scleromyositis (Anti-PM/Scl 75 and 100) characterized by sclerodactyly, calcinosis, telangiectasia and myositis, reported a slow-growing lump in the right thigh. A CT scan showed multiple calcifications and a fluid collection with a calcific rim in the vastus medialis, suggestive of ‘milk of calcium’ calcinosis (Fig. 1a–d). The patient was treated with MTX for 4 years, then discontinued for clinical stability. When she turned 23 years old, she complained of diffuse, mild muscle pain, with a slight increase in creatine phosphokinase levels. Thus, she underwent a whole-body MR which demonstrated the persistence of fluid collection with calcific rim (Fig. 1e–g) and increased soft tissue calcifications in the extremities.

**Figure 1.**
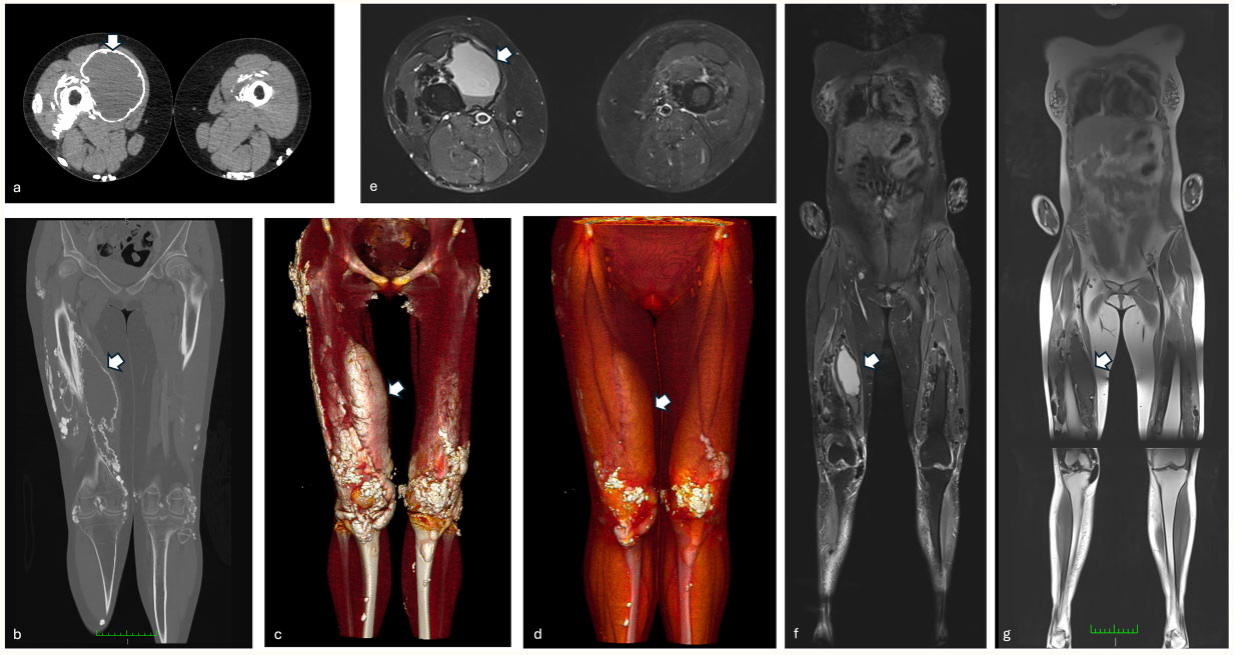
CT and MR features of milk of calcium calcinosis in scleromyositis. Leg axial CT (**a**) of a 15-year-old female with scleromyositis showing a fluid collection with a calcific rim in the right vastus medialis, corresponding to the clinically reported slow-growing mobile lump. Coronal (**b**) and 3D (**c** and **d** ) reconstructions show the extent and the calcific rim of the milk of calcium lesion. Axial short inversion time inversion recovery (STIR) (**e**), coronal STIR (**f** ) and T1-weighted (**g** ) whole-body MR, performed 8 years later, nicely demonstrating the fluid content and the peripheral calcific margins of the lesion that remained stable over time. Multiple soft tissue calcifications are visible on CT (as hyperdense areas) and MR (as signal voids) as part of the calcinosis pattern which increased over time

Scleromyositis is an overlap syndrome that has not been completely characterized. The so-called ‘milk of calcium’ is a rare type of calcinosis, and only a few cases have reported its imaging features [1, 2]. Unfortunately, there is no effective therapy for calcinosis, with anecdotal reports about Janus kinase (JAK) inhibitors for myositis.

To the best of our knowledge, this is the first case showing MR and CT imaging of the ‘milk of calcium’ calcinosis in scleromyositis over time, demonstrating that both are suitable tools for assessing this rare and disabling manifestation of the disease.

## Data Availability

The corresponding author (C.G.) is available to share images and anonymized clinical history on request.
